# Is it really keratoconus or pseudo-keratoconus? – Topographic mimicry

**DOI:** 10.1007/s00417-025-07067-2

**Published:** 2026-04-10

**Authors:** Agata Anna Wykrota, Berthold Seitz, Elias Flockerzi

**Affiliations:** Department of Ophthalmology, Saarland University Medical Center (UKS), Kirrberger Straße 100, Building 22, 66421 Homburg/Saar, Germany

**Keywords:** Cornea, Pseudokeratoconus, Keratoconus, Corneal topography

## Abstract

**Purpose:**

This case series aimed to present eyes with a topographic pseudo-keratoconus configuration resulting from corneal diseases other than keratoconus (KC).

**Methods:**

Inclusion criteria were a topographic classification of the cornea as KC by Scheimpflug imaging, considering the topographic keratoconus classification (TKC), and a typical height-data profile with irregular astigmatism and an inferior-superior asymmetry value (I-S value) > 1.4 at 6 mm of the cornea. Clinical slit-lamp examination, best-corrected visual acuity, and Scheimpflug tomography (Pentacam^®^, OCULUS, Wetzlar, Germany) were analyzed. Standard deviations and 95% confidence intervals were added where applicable to enhance descriptive precision.

**Results:**

Fifty-seven eyes of 37 patients were included (mean age 49 years, range 18–78; female-to-male ratio 16:21). The mean Kmax was 50 D (42.4–57.8), mean TKC 2, and I-S value 2.87 (1.4–4.91). Mean corneal thickness at the thinnest point was 528 μm (274–709). Study eyes were divided into three groups according to the underlying cause of topographic change: (1) inferior steepening (e.g. Map-Dot-Fingerprint dystrophy); (2) superior configuration (e.g. dry-eye disease or Salzmann nodular degeneration); and (3) newly developed keratoconus-like configuration after phototherapeutic keratectomy.

**Conclusions:**

Different corneal pathologies can imitate a topographic KC configuration. Differentiating pseudo-keratoconus from true ectasia is clinically relevant to avoid inappropriate treatment and ensure correct patient counseling. Identifying and distinguishing these pathologies – particularly at the slit lamp – are essential for accurate diagnosis and adequate therapy.

## Key Message

- What is known
  - Keratoconus is a bilateral, quasi-inflammatory progressive ectatic corneal disease with paracentral thinning, which, if overlooked,may lead to visual impairment.
  - A diagnosis of keratoconus can be made using slit-lamp examination, Scheimpflug tomography, and analysis of cornealbiomechanics.
- What is new
  - There are many corneal disease entities, that can mimic keratoconus topographically, showing ectasia and paracentral, inferior-temporal thinning.
  - Most eyes with a pseudokeratoconic pattern in this study presented with a corneal scar or Map-Dot-Fingerprint dystrophy.

## Introduction

Keratoconus (KC) is a bilateral, quasi-inflammatory progressive ectatic corneal disease with paracentral thinning [1–4]. Science has not succeeded in unraveling the mystery surrounding the cause of KC and its related forms. Apparently, there are certain genetic predispositions, but also exogenous factors, such as excessive eye rubbing, seem to play a major role. Often KC occurs associated with other diseases or syndromes, e.g. neurodermatitis, allergies and trisomy 21 [5]. As a result, a patient with KC may present with progressive irregular astigmatism, scarring and visual impairment [6]. Therapeutic options depend on the progression of the disease and include contact lens fitting or surgical interventions, like cross-linking, intracorneal ring segments (ICRS) or keratoplasty (deep anterior lamellar keratoplasty = DALK or penetrating keratoplasty = PKP) [7]. Ectatic corneal diseases are not that rare with an average of at least one patient per 2,000 inhabitants in Europe and can be diagnosed both clinically and by means of special imaging techniques. In early stages, they are hardly recognizable and have been therefore often “overlooked” in the past. A reliable diagnosis of early KC cases based on slit lamp examination alone is impossible. For this purpose, a computer-based, two-dimensional curvature image is necessary, the so-called corneal tomography analysis (also known as Scheimpflug-imaging or anterior segment OCT). Thus, certain parameters such as focal corneal steepening, increased astigmatism, abnormally increased anterior or posterior elevation can show an ectatic pattern [8]. Nowadays, tomography has begun to supplement topography which has been the gold-standard in the diagnosis of keratectasia over the last decades. Tomography in turn is supplemented by the analysis of corneal biomechanics that can be done based on the analysis of the deformation response of the human cornea to a standardized air puff indentation generated by the Corvis^®^ ST (ST, OCULUS, Wetzlar, Germany). This device aims to distinguish between healthy and KC corneas based on the biomechanical deformation response of the cornea [6, 9]. Instrumental misdiagnosis of KC may include poor fixation, tear film instability, loss of corneal transparency and corneal dystrophies or degenerations. Furthermore, there are other disease entities, that can mimic KC topographically, showing an ectasia and paracentral, inferior-temporal thinning [10].

In clinical practice, corneal topography is typically performed when patients present with reduced or fluctuating vision, irregular astigmatism on refraction, or slit-lamp signs such as scarring or epithelial irregularities that warrant topographic confirmation. This reflects the routine diagnostic pathway in which imaging is selectively indicated based on clinical findings. Corneal topography was long considered the gold standard for diagnosing corneal ectasia, and even today, ophthalmologists, optometrists, and opticians may still primarily look at topographic patterns and then refer patients to centers if they find any abnormal patterns. Differentiating pseudo-keratoconus from true ectasia is clinically relevant to avoid unnecessary cross-linking or inappropriate contact-lens prescriptions and to ensure proper patient counseling.

The aim of this case series is to present eyes with a topographic KC configuration, but actually no KC exists after careful slit lamp examination and analysis of tomography and corneal biomechanics, thus leading to the diagnosis of a so-called “pseudo-keratoconus” [11, 12].

## Materials and methods

In 2010, the Department of Ophthalmology at the Saarland University Medical Center in Homburg/Saar established the Homburg Keratoconus Center (HKC) to conduct research on the causes, early diagnosis and the individual, stage-dependent therapy of KC. It includes currently more than 3,400 KC patients [13].

According to Belin, there are some findings, which are mandatory to diagnose KC: abnormal posterior elevation, abnormal corneal thickness distribution, clinical non-inflammatory corneal thinning, and tomography (Scheimpflug or OCT = optical coherence tomography), currently considered the best diagnostic test to detect early KC. Another helpful parameter might be a maximal anterior keratometry (Kmax) of greater or equal to 47.0 diopters (dpt) [14].

Inclusion criteria for the present study were a classification of the cornea as KC by Scheimpflug imaging using the topographic keratoconus classification (TKC) and a typical elevation data profile with irregular astigmatism and inferior-superior asymmetry value (I-S value) of more than 1.4 at 6 mm (3 mm radii) of the cornea. The differentiation between KC and other corneal pathology was based on the patient’s medical history, clinical findings on the slit lamp and corneal thickness.

The clinical image in the slit lamp examination (40x magnification), the best-corrected visual acuity and Scheimpflug tomography of the anterior segment of the eye (Pentacam^®^, OCULUS, Wetzlar, Germany) as well as the corneal biomechanics (Corvis^®^ ST, OCULUS, Wetzlar, Germany) using the Corvis Biomechanical Index (CBI), Tomographic Biomechanical Index (TBI) and biomechanical E-staging were analyzed [15]. However, the corneal biomechanics measurements were not carried out in all patients, since slit-lamp examination often already allowed a diagnosis other than keratoconus to be made beforehand.

Scheimpflug imaging was performed by certified technicians following a standardized acquisition protocol (adequate blinking, good fixation, and repeated scans until image quality met manufacturer standards) to minimize pseudo-keratoconic artefacts. In advanced cases, a score “model deviation” had to be tolerated.

Corneal biomechanics were obtained in 11 of 57 eyes and evaluated descriptively. Because diverse corneal pathologies (e.g. scarring, Fuchs endothelial corneal dystrophy, Salzmann nodular degeneration) can influence the corneal deformation response, these data were used qualitatively to support morphological interpretation rather than for statistical comparison between groups.

Continuous variables are reported as mean ± standard deviation (SD) or median [range], and categorical data as n/N (%). Where relevant, 95% confidence intervals (CIs) were calculated using the Wilson method. Inferential testing was not performed owing to the descriptive, retrospective design.

The local ethics committee of Saarland (Ethikkommision bei der Ärztekammer des Saarlandes) was informed about the study and no ethical approval was required according to the committee. This study was conducted in accordance with the Declaration of Helsinki and the institutional guidelines. Patient identity was masked in all information tables and images.

## Results

In our case series, 57 eyes of 37 patients were included. The mean age was 49 (18–78) years, and the female-to-male ratio was 16 to 21. The mean best-corrected Snellen visual acuity was 20/40 (20/2000-20/20). The mean flat keratometry (K1) amounted to 42.9 (36.6–49.3) diopters, the mean steep keratometry (K2) 46.1 (38.8–63.7) diopters, and the mean maximum single point keratometry (Kmax) 50 (42.4–57.8) diopters. The mean anterior radius of curvature (ARC) at 3 millimeters was 8.29 (6.84–10.62), the mean posterior radius of curvature (PRC) at 3 mm 6.89 (3.44–7.05), the mean TKC stage was 2, and the I-S value 2.87 (1.4–4.91). The mean corneal thickness at the thinnest point was 528 (274–709) µm, whereas the mean astigmatism was 3.11 (0.1–10.6) diopters (Table 1).

**Table 1:** Additional details of the study eyes with pseudokeratoconic topography

Study eyes *N* = 57	Mean value (if available)
Age	49 (18–78) years
Gender	Male 21; Female 16
Best-corrected visual acuity (Snellen)	20/40 (20/2000-20/20)
K1	42.9 (36.6–49.3) dpt
K2	46.1 (38.8–63.7) dpt
Kmax	50 (42.4–57.8) dpt
ARC (3 mm)	8.29 (6.84–10.62)
PRC (3 mm)	6.89 (3.44–7.05)
TKC value	2 (0–4)
I-S value	2.87 (1.4–4.91)
Corneal thickness	528 (274–709) µm
Astigmatism	3.11 (0.1–10.6) dpt

*K1* flat keratometry, *K2* steep keratometry, *Kmax* maximum single-point keratometry, *ARC* anterior radius of curvature, *PRC* posterior radius of curvature, *TKC* topographic keratoconus classification, *I-S* value – inferior-superior asymmetry value

Descriptive statistical parameters are presented as mean ± standard deviation (SD), with 95% confidence intervals (CIs) added where appropriate to highlight variability within subgroups.

The study eyes were divided into three groups according to the underlying cause of the topographic changes. The first group (*n* = 35) included corneal diseases with inferior steepening in topography (corneal scar *n* = 17, Map-Dot-Fingerprint dystrophy *n* = 10, Fuchs endothelial corneal dystrophy (FECD) *n* = 5, vortex keratopathy *n* = 2, Salzmann nodular degeneration *n* = 1).

The second group (*n* = 19) showed a superior steepening that could be interpreted as a superior keratoconus configuration (Map-Dot-Fingerprint dystrophy *n* = 7, irregular astigmatism without other corneal pathology *n* = 4, dry eye disease (DED) *n* = 4, herpetic corneal scar *n* = 2, Salzmann nodular degeneration *n* = 2).

A small, third group included eyes with postoperative newly occurring KC configuration after phototherapeutic keratectomy (*n* = 3).

In terms of subgroup comparison, among eyes with inferior steepening (*n* = 35), Map-Dot-Fingerprint dystrophy represented 10/35 (28.6%; 95% CI 16.3–45.1). Among eyes with superior steepening (*n* = 19), the same dystrophy represented 7/19 (36.8%; 95% CI 19.1–59.0). Salzmann nodular degeneration was the underlying diagnosis exclusively in superior or central irregularities.

Among the eyes with available corneal biomechanics (11/57), deformation amplitude ranged from 1.05 to 1.27 mm, consistent with a stiffened corneal response rather than true ectasia.

**Fig. 1:**
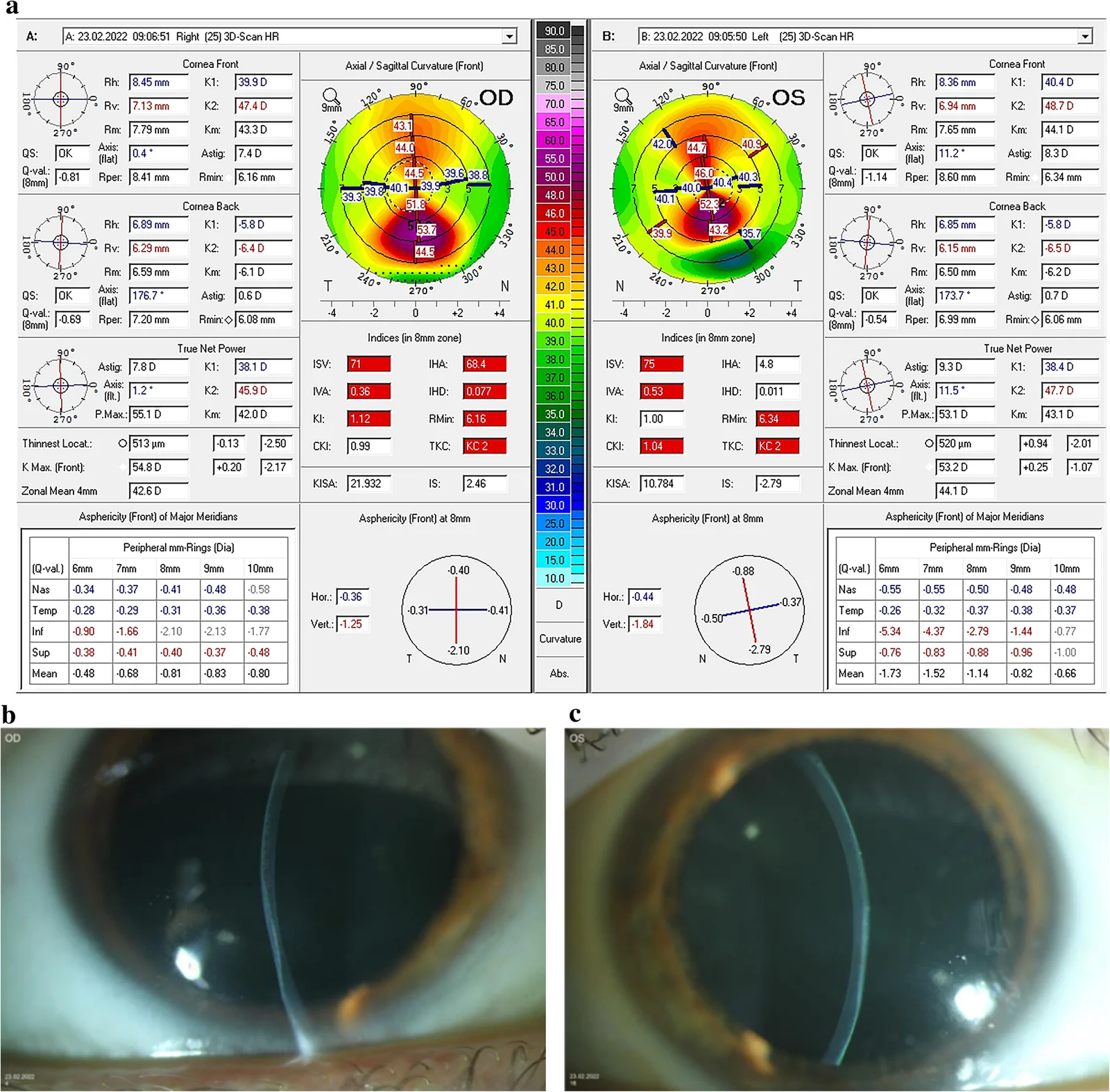
**a** Scheimpflug topography of the anterior segment of the eye in a patient with bilateral inferior corneal scars as a result of resolved incomplete lid closure following a traffic accident. Red-marked fields indicate topographic keratoconus indices suggesting keratectasia. Topographic keratoconus classification: TKC 2 keratoconus stage for both eyes. **b** Photograph displaying corneal scarring on the right eye as a result of resolved incomplete lid closure following a traffic accident. **c** Photograph displaying corneal scarring on the left eye as a result of resolved incomplete lid closure following a traffic accident

We present a case of a 38-year-old male patient (Fig. 1a-c) who came to our department with a suspected case of KC and the subjective reduction of visual acuity since a car accident 20 years earlier. The BCVA was 20/30 on the right eye (OD) and 20/20 on the left eye (OS) and intraocular pressure was 10 mmHg on both eyes. The patient presented with hyperopia, with a sphere of + 3.25 on OD and + 2.50 on OS, as well as astigmatism of -7.50 on OD and − 8.25 on OS. Tomography showed the corneal thickness at the thinnest point of 513 μm on OD, and 520 μm on OS, inferior corneal steepening with Kmax of 54.8 dpt (OD) and 53.2 (OS) as well as TKC and I-S values of 2 and 2.46 on both eyes respectively (Fig. 1a). However, there was visible inferiorly located corneal scarring (Fig. 1b-c). There were no further clinical and diagnostic ocular abnormalities, and we therefore diagnosed corneal scarring with highly irregular astigmatism as a result of resolved incomplete lid closure following a traffic accident and recommended fitting of rigid gaspermeable contact lenses.

**Fig. 2:**
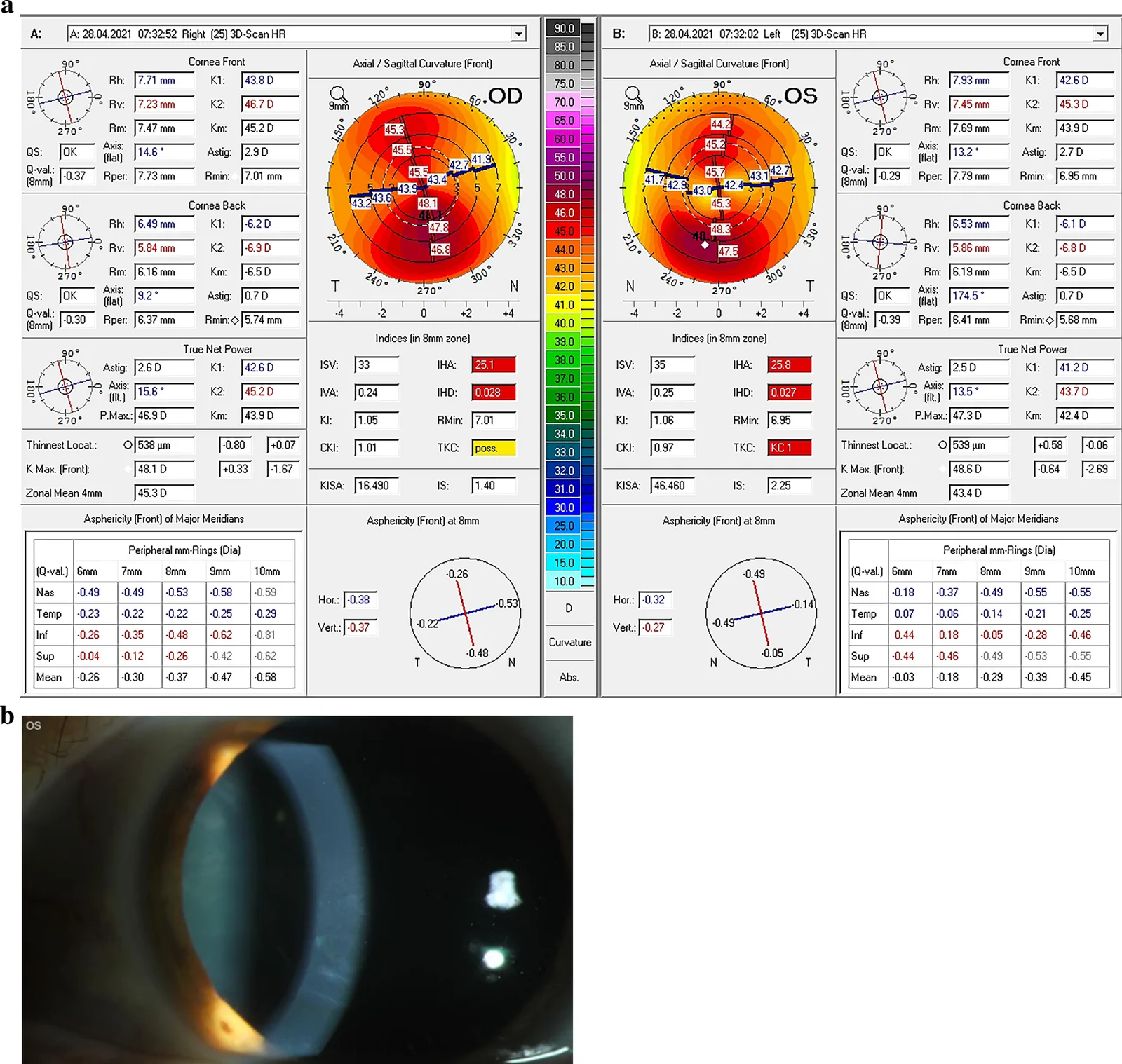
**a** Scheimpflug topography of the anterior segment of the eye in a patient with bilateral Map-Dot-Fingerprint corneal dystrophy. Red-marked fields indicate topographic keratoconus indices suggesting keratectasia. Topographic keratoconus classification: possible keratoconus in the right eye and TKC 1 keratoconus stage in the left eye. **b** Photograph displaying superficial grey shapes characteristic of Map-Dot-Fingerprint corneal dystrophy

Another example is the case of a 64-year-old female patient (Fig. 2a-b) who presented with suspected case of KC and the subjective fluctuative reduction of visual acuity for the past months. The BCVA was 20/30 on the right eye (OD) and 20/50 on the left eye (OS) and intraocular pressure was 13 mmHg on OD and 14 mmHg on OS. The patient had no major refractive error on both eyes. Tomography showed the corneal thickness at the thinnest point of 538 μm on OD, and 539 μm on OS, inferior corneal steepening with Kmax of 48.1 dpt (OD) and 48.6 (OS) as well as TKC values of “KC possible” on OD and 1 on OS, with the I-S value of 1.4 on both eyes (Fig. 2a). A detailed slit-lamp examination in retroillumination revealed superficial grey shapes and fingerprints (Fig. 2b). Thus, we diagnosed this patient with Map-Dot-Fingerprint dystrophy and performed a phototherapeutic keratectomy as the patient had disturbing fluctuation of visual acuity.

**Fig. 3:**
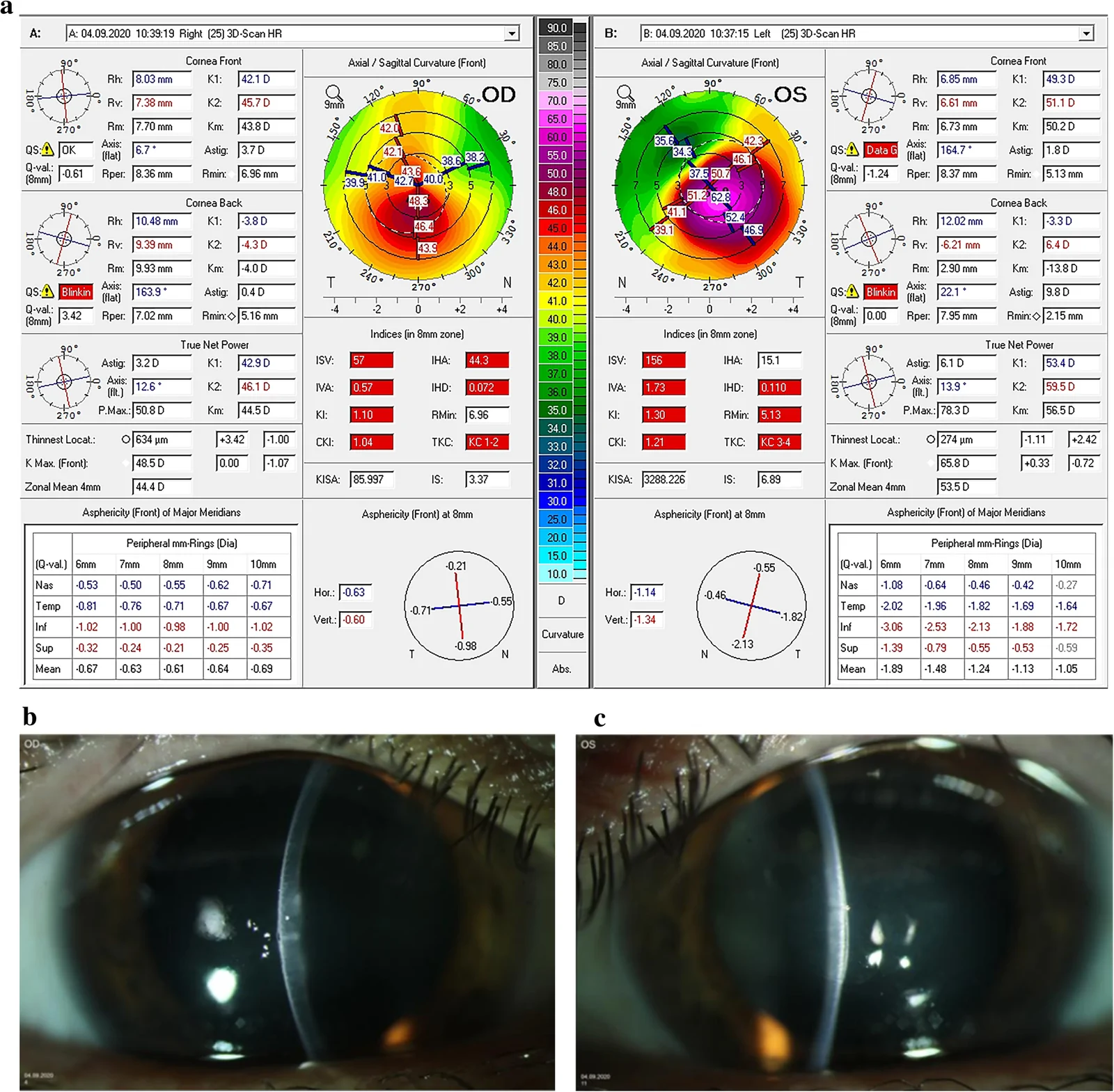
**a** Scheimpflug topography of the anterior segment of the eye in a patient with bilateral Fuchs endothelial corneal dystrophy. Red-marked fields indicate topographic keratoconus indices suggesting keratectasia. Topographic keratoconus classification: TKC 1–2 in the right eye and TKC 3–4 in the left eye. **b** Photograph displaying corneal oedema with mini-bullae on the right eye in a patient with Fuchs endothelial corneal dystrophy. **c** Photograph displaying corneal oedema with large bullae on the left eye in a patient with Fuchs endothelial corneal dystrophy

Another case is a 44-year-old male patient (Fig. 3a-c) who came to our department with suspected corneal edema after acute hydrops and the subjective reduction of visual acuity as well as increased lacrimation on the left eye more than on right eye in the past 3 to 4 months. The BCVA was 20/50 on the right eye (OD) and 20/60 on the left eye (OS) and intraocular pressure was 13 mmHg on OD and 12 mmHg on OS. The patient presented with myopia, with a sphere of -2.00 on both eyes, as well as astigmatism of -1.00 on both eyes. Tomography showed the corneal thickness measurement at the thinnest point of 634 μm on OD and 274 μm on OS, inferior corneal steeping with Kmax of 48.5 dpt (OD) and 65.8 (OS) as well as TKC values of 1–2 on OD and 3–4 on OS, with the I-S value of 3.37 on both eyes (Fig. 3a). However, there were visible guttae, corneal edema and folds in the Descemet’s membrane (Fig. 3b-c). There were no further clinical and diagnostic ocular abnormalities. FECD was diagnosed and Descemet Membrane Endothelial Keratoplasty (DMEK) was performed subsequently on both eyes.

## Discussion

Keratoconus is a progressive ectatic corneal disease that may be diagnosed using Scheimpflug tomography of the anterior segment of the eye (Pentacam^®^, OCULUS, Wetzlar, Germany) and the analysis of corneal biomechanics (Corvis ST^®^, OCULUS, Wetzlar, Germany). Additionally, it is important to obtain a proper patient anamnesis and run a careful slit lamp examination to search for clinical KC signs of paracentral stromal thinning, Fleischer’s ring, Vogt’s striae and even corneal scarring which can lead from mild to considerable impairment of vision and, consequently, quality of life. Thus, proper diagnosis and treatment are of great significance in order to stop progression, reduce the astigmatism, or get rid of the corneal scar e.g. by means of anterior lamellar or penetrating keratoplasty. However, in case of a topographic KC configuration with no obvious signs of KC on slit lamp examination, a so-called “pseudo-keratoconus” should be considered [11]. At the time, there are only few publications on the possible causes of such topographic patterns.

According to Sideroudi et al. [12], the most relevant corneal pathologies that may imitate the topographic KC pattern are keratoglobus, posterior KC, pellucid marginal degeneration and Fuchs-Terrien marginal degeneration. The current study showed that out of a total of 57 included eyes, the following corneal pathologies could lead to a pseudokeratoconic configuration as well: corneal scar in 19 eyes, Map-Dot-Fingerprint dystrophy in 17 eyes, FECD in 5 eyes, irregular astigmatism without other corneal pathology in 4 eyes, DED in 4 eyes, Salzmann nodular degeneration in 3 eyes, status after phototherapeutic keratectomy in 3 eyes and vortex keratopathy in 2 eyes.

In contrast to previous series or case reports describing isolated causes of pseudo-keratoconus, the current analysis combines a comparatively large cohort with quantitative topographic data and morphological correlation. This permits a broader overview of the relative frequency of mimicking entities within the same institutional population.

Corneal scarring may be found in eyes after infections or trauma and can lead to visual impairment through blocked or scattered light and scar-induced irregular astigmatism [11]. In fact, 19 of 57 of our study eyes with a pseudokeratoconic pattern presented with a corneal scar. Stromal herpetic keratitis typically results in stromal corneal scarring and thinning with induced irregular astigmatism [16, 17], as can be seen in 6 of 19 eyes in our study. Interestingly, chronic ocular rosacea can also lead to an induced KC pattern as a result of chronic exposure of the inferior cornea to inflammatory and matrix-degrading factors in the inferior tear meniscus with subsequent inferior corneal thinning and high astigmatism [18, 19]. Furthermore, an incomplete lid closure can result in corneal scarring and an irregular astigmatism and, thus, a pseudokeratoconic pattern in topography (Fig. 1a-c).

Another corneal pathology possibly leading to the topographic misdiagnosis of KC is epithelial basement membrane dystrophy, also known as Map-Dot-Fingerprint dystrophy. It is characterized by an aberrant and multi-layered basement membrane which causes recurrent corneal erosions and an irregular astigmatism in advanced cases [20]. As a result, eyes with this entity can present with a pseudokeratoconus pattern, as seen in 17 eyes in the present study (Fig. 2a-b). Wardani et al. similarly described an inferior inhomogenous epithelial thickening as a possible contributing factor in the appearance of suspicious KC-like topographies in 12 eyes [10].

FECD can be usually diagnosed clinically on the slit lamp examination because of its typical appearance with guttae, Descemet’s membrane thickening and consecutive epithelial and stromal corneal edema [21]. However, the presence of corneal edema with bullae can result in a focal elevation of the anterior corneal surface. As such, topographically the cornea seems to have an irregular astigmatism and inferior steepening, thus mimicking KC (Fig. 3a-c), which was seen in 5 eyes in our study.

Although an irregular astigmatism is usually associated with KC, it can also be present in normal human eyes as an isolated entity without the presence of KC (as seen in 4 eyes in this study) [22]. KC can be excluded in patients with an irregular astigmatism using the tomographic and biomechanical ABCDE KC staging. It provides analysis of the anterior (A) and the posterior (B) corneal curvature (in an optical zone of 3 mm centered on the thinnest corneal point), the thinnest corneal thickness (C), the best spectacle-corrected visual acuity (D), and corneal biomechanics (E) [6]. However, a patient could have a KC classification of A0B0C0D0E0 and still have keratoconus (as in forme fruste KC), and as such patients with an irregular astigmatism without any other corneal pathology should be observed during follow-up.

Further corneal pathology associated with a topographic pseudokeratoconus configuration in this study is Salzmann nodular degeneration. It is characterized by the presence of noninflammatory, grey, elevated superficial and subepithelial nodules which, at the presence of progression, may result in a decreased surface regularity, recurrent erosions, and visual impairment [23]. Eyes with inferiorly located nodules can show an inferior steepening in topography, thus mimicking KC [24].

A pseudokeratoconus configuration was present in 3 examined eyes with a history of phototherapeutic keratectomy, which was also described by Chorny et al. [24]. It is most likely to be associated with postoperatively induced astigmatism by stromal remodeling.

Lastly, 4 eyes with DED and 2 eyes with vortex keratopathy showed a topographic pseudokeratoconus pattern in our study. It could be due to increased tear film osmolarity and levels of inflammatory mediators in tears with subsequent surface irregularity in the former [25] and amiodarone drug deposition in the corneal epithelium with its secondary thickening leading to irregular astigmatism in the latter [26].

An important factor is an adequate alignment during the acquisition of corneal topography / tomography, since rotational misalignment or poor fixation may induce corneal asymmetry on elevation and curvature maps as well as pseudokeratoconus patterns (Hick et al.) [24, 27]. Therefore, the aforementioned should be taken into consideration when analysing a topography with KC pattern.

Recent advances in anterior-segment OCT (AS-OCT) and artificial intelligence further refine the differential diagnosis of KC. AS-OCT epithelial thickness mapping (ETM) characterizes the epithelial remodeling that often masks stromal ectasia and helps distinguish true KC from ‘pseudo-keratoconus’ secondary to surface or lens-related effects. In particular, OCT-derived epithelial/curvature maps and ETM-based indices have been shown to differentiate contact-lens warpage and other non-ectatic irregularities from KC – even at subclinical stages – by revealing discordant patterns of epithelial thinning/thickening relative to topographic steepening [28]. In addition, OCT-based grading frameworks (e.g., STEP), which integrate epithelial and stromal metrics, show good agreement with the tomographic ABCD/BEST staging and may be useful when Scheimpflug tomography suggests KC but slit-lamp findings are equivocal, as in several entities reported in our cohort [6, 29].

Concurrently, AI models trained on corneal tomographic maps and multimodal metrics (including biomechanical/tomographic indices) achieve high accuracy for detecting early or asymmetric KC; nonetheless, current reviews stress the need for robust external validation and harmonized datasets before routine deployment. These tools could complement clinical examination and imaging to reduce false-positive ‘KC-like’ classifications in eyes with scars, epithelial basement membrane dystrophy, FECD-related edema, or tear-film instability, as observed in our series [30].

To the best of our knowledge, this is the largest study examining eyes with a topographic “pseudo-keratoconus” and its possible clinical causes. The scientific originality of the work lies in the systematic comparison of different mimicking pathologies within a single institutional cohort, combining corneal topography and in part biomechanics to refine diagnostic interpretation. The main limitation of the study is its retrospective design and the absence of longitudinal follow-up data. Nevertheless, the present analysis represents one of the largest single-centre cohorts reported to date that systematically compares the different corneal pathologies capable of resulting in a topographic pseudo-keratoconus pattern. Prospective studies could be helpful to confirm these findings and to further elucidate the diagnostic boundaries between true and pseudo-ectatic disease. For clinical practice, we recommend: (1) confirming any keratoconus-like topography by careful slit-lamp inspection for scarring, dystrophy or oedema; (2) repeat imaging after optimisation of the tear film and fixation; (3) use epithelial mapping or anterior-segment OCT when tomography and clinical findings are discordant; and (4) defer keratoconus-specific interventions, such as cross-linking, until true ectasia is verified.

## Conclusions

In conclusion, this case series shows that various corneal pathologies can exhibit a KC configuration by means of corneal tomography. Most of examined eyes with a pseudokeratoconic pattern presented with a corneal scar and Map-Dot-Fingerprint dystrophy. This study represents one of the largest single-centre cohorts systematically characterizing pseudo-keratoconus configurations and highlights the diagnostic overlap between different corneal disorders. Recognising these pseudo-ectatic patterns is clinically relevant to prevent misdiagnosis, avoid unnecessary cross-linking procedures, and ensure appropriate patient counselling and treatment of other underlying corneal diseases. The identification – not least at the slit lamp – and differentiation of these pathologies from KC are important not only for adequate patient counseling in daily practice, but also for further treatment and adequate therapy.
